# Factors Associated with Atypical Cognitive Trajectories in Older Adults with Down Syndrome

**DOI:** 10.1002/alz70857_105703

**Published:** 2025-12-25

**Authors:** Diana K. Valencia, Wayne Silverman, Sigan L Hartley, Shahid Zaman, Adam Brickman, Florence Lai, Mark Mapstone, Sharon J. Krinsky‐McHale, Jordan P. Harp, Patrick J. Lao, Ira T. Lott, H. Diana Rosas, Benjamin L Handen, Bradley T Christian, Beau Ances, Frederick A. Schmitt, Christy L. Hom

**Affiliations:** ^1^ University of California, Irvine, Irvine, CA, USA; ^2^ Waisman Center, University of Wisconsin‐Madison, Madison, WI, USA; ^3^ University of Cambridge, Cambridge, United Kingdom; ^4^ Columbia University, New York, NY, USA; ^5^ Harvard/Massachusetts General Hospital, Boston, MA, USA; ^6^ New York State Institute for Basic Research in Developmental Disabilities, Staten Island, NY, USA; ^7^ University of Kentucky / Sanders‐Brown Center on Aging, Lexington, KY, USA; ^8^ Taub Institute for Research on Alzheimer's Disease and the Aging Brain, New York, NY, USA; ^9^ Massachusetts General Hospital, Harvard Medical School, Boston, MA, USA; ^10^ University of Pittsburgh, Pittsburgh, PA, USA; ^11^ Department of Medical Physics, University of Wisconsin, Madison, WI, USA; ^12^ Washington University, St. Louis, MO, USA; ^13^ Sanders‐Brown Center on Aging Spinal Cord & Brain Injury Research Center, University of Kentucky, Lexington, KY, USA

## Abstract

**Background:**

90% of adults with Down syndrome (DS) will develop Alzheimer's dementia (AD) in their lifetime, the majority within 3 years of symptom onset. However, clinicians need to be confident that declines are not temporary or due to treatable causes before diagnosing prodromal AD. The present study investigates the medical, psychiatric, and life factors associated with atypical cognitive decline in DS, a population with genetic risk for AD. We aim to identify factors that contribute to temporary or non‐progressing cognitive decline over a three‐year period.

**Method:**

Thirty‐eight adults with DS (ages 43 to 62) from the Alzheimer Biomarker Consortium in Down Syndrome (ABC‐DS) were classified as having prodromal AD (MCI‐DS) at one or more time points. AD clinical status was determined through a consensus process using only clinical and cognitive data. There were three trajectories based upon longitudinal data: (1) progressors (MCI‐DS to dementia), (2) reverters (MCI‐DS to cognitively stable), or (3) non‐progressors (MCI‐DS for 3 time points). Comorbidities and life stressors were collected through study partner interviews. Baseline amyloid PET scans and plasma AD biomarkers are used in the present study.

**Result:**

Using chi‐square and Kruskal‐Wallis tests, non‐progressors (*n* = 6, M_age_ = 48.67) were younger (*p* = .032), had lower centiloid values (*p* = .002) and had higher prevalence of seizure disorder (*p* = < .001), hearing impairment (*p* = < .001), and a friend move away (*p* = .027) than progressors (*n* = 26, M_age_ = 52.88). Reverters (*n* = 6, M_age_ = 49.17) had a higher incidence of psychotic disorder (*p* = .028) compared to non‐progressors and progressors. Both atypical groups had lower incidence of COVID infection (*p* = .046), but still had high amyloid load (M=30.50CL and 48.02CL, respectively).

**Conclusion:**

In our sample of adults with DS and MCI‐DS, 31.6% did not progress to dementia despite having high levels of amyloid PET. Those whose decline plateaued had more medical conditions and life stressors. Those with temporary decline had more psychiatric problems. Primary care physicians need to understand which reversible or treatable factors are most likely to lead to a misdiagnosis of prodromal AD and address them accordingly.

